# Parents’ knowledge of emergency management of avulsed permanent teeth in children and adolescents in the State of Qatar: a questionnaire cross-sectional study

**DOI:** 10.1007/s40368-023-00829-8

**Published:** 2023-08-31

**Authors:** M. Al Sheeb, F. A. Al Jawad, H. Nazzal

**Affiliations:** 1 Hamad Dental Centre, Hamad Medical Corporation, Doha, Qatar; 2 College of Dental Medicine, Qatar University, Doha, Qatar

**Keywords:** Avulsion, Parental knowledge, Questionnaire, Storage medium, Tooth replantation

## Abstract

**Purpose:**

To elucidate the level of parental knowledge in Qatar regarding the management of avulsed teeth.

**Methods:**

A self-administered questionnaire was completed by 400 parents attending their children’s routine dental appointments. The questionnaire comprised of items on sociodemographics, parents’ prior knowledge on management of avulsion, parents’ willingness to replant avulsed teeth at the site of injury, and parents’ opinion as to the best format for future parental education on the management of dental trauma. Univariate and multivariate logistic regressions were employed to assess the association between sociodemographic status and knowledge in the management of avulsion.

**Results:**

The overall mean score of parents’ knowledge was (23%). Only 23.3% (*n* = 93) of parents thought that it was possible to reinsert an avulsed permanent tooth. Out of those, only 12.9% (*n* = 12) indicated willingness to reinsert the tooth back into its socket, while 48.4% (*n* = 49) indicated that an avulsed tooth should be stored using physiological media. Less than one third (27.8%) indicated that they would seek professional help within 30 min. Males were less likely to give favourable answers when compared to females (OR = 0.43, 95% CI = 0.22–0.84). Age groups “31–40” years and “41–50” years were more likely to give favourable answers when compared to 20–30 years age group (OR = 2.8, 95% CI = 1.05–8.0and OR = 3.8, 95% CI = 1.3–11.48; respectively).

**Conclusion:**

This study highlights critical deficiencies in parental knowledge on the management of tooth avulsion and the need to improve parents’ knowledge by developing easily accessible onsite emergency management tools.

## Introduction

Traumatic dental injuries occur frequently in children and adolescents cross the world with a wide prevalence rate ranging between 4.9 and 37% (Ozer et al. 2012). Permanent tooth avulsion: complete detachment of tooth from the socket is the most complicated and serious type of dental trauma (Andreasen et al. 2019). It comprises 1–16% of dental injuries, with peak incidence in 7–11-year-old children affecting mainly the maxillary central incisors (Andreasen et al. 2019).

Prompt and appropriate onsite intervention of avulsed teeth is crucial for the success and survival of affected teeth. Ideally, an avulsed tooth should be immediately replanted into its socket to avoid further damage to the periodontal ligament cells (Fouad et al. 2020). Studies have shown that the prognosis of avulsed teeth depends on many factors including time elapsed between trauma and replantation, the type and condition of storage medium, the stage of root formation and the presence of contamination (Santos et al. 2009). Andreasen et al. (2019) reported that survival rates of avulsed teeth range between 39 and 89% with higher success rates of teeth managed appropriately especially at the site of injury.

Storage media have a critical role in the survival of periodontal ligament cells (Fouad et al. 2020). While dry storage causes an irreversible injury to the periodontal ligament cells, resulting in loss of the replanted tooth over time, wet media differ in their role. Milk, which is readily available and accessible at the place of injury, has a favourable osmolality and composition for the viability of periodontal ligament cells, therefore, has been recommended for temporary storage of avulsed teeth (Blomlöf 1981; Blomlöf et al. 1983). While water is not recommended, as a result of its low osmolality, the use of patient’s own saliva could be used for short storage periods (Cvek et al. 1974; Andreasen 1981; Andreasen et al. 1995). The use of especially composed cell culture media has been recommended in the literature; however, such media are seldom accessible at the place of accident (Andreasen et al. 1995; Chappuis and von Arx 2005; Andreasen et al. 2019).

Parents are usually present at the site of injury; therefore, understanding their level of knowledge on the appropriate management of avulsed teeth and their willingness to replant these teeth in a timely manner are of great importance in improving the long-term prognosis of avulsed teeth. Such area has never been assessed in the State of Qatar where the culture and sociodemographic structure are unique and different to that of neighbouring and international countries. Therefore, the aim of this study was to elucidate the level of parental knowledge with respect to the management of avulsed teeth and willingness to replant them in the State of Qatar. Furthermore, parent’s preference of the means of obtaining such knowledge has been assessed.

### Materials and methods

The study was presented in accordance with the Strengthening the Reporting of Observational Studies in Epidemiology statement (STROBE).

This was a cross-sectional study where ethical approval was obtained from the Medical Research Center (MRC) at Hamad Medical Corporation (HMC), Doha, Qatar (MRC-01-19-086).

Parents of children attending the paediatric dental department at Hamad Dental Centre, Hamad Medical Corporation, Doha, Qatar, for their children’s routine dental appointments were invited to take part in this study. Parents, able to read and write Arabic, were consecutively recruited by approaching them to take part in this study. Informed consent was obtained prior to asking the participants to complete the anonymous study questionnaire while waiting for their child’s appointment. Data collection was performed between April 2019 and February 2020.

The primary outcome of this study was the level of parental knowledge with respect to the management of avulsed teeth. Sociodemographic variables were considered predictors (independent variables), which included gender, age, education level, and income. Parents willingness to replant avulsed teeth at the site of injury and their preference on the method of obtaining appropriate management information were also sought as secondary outcomes.

A 16-item hard copy questionnaire was constructed based on previous studies that investigated parental previous experiences and awareness of different aspects of the emergency management of avulsed permanent teeth (Al-Jame et al. 2007; Santos et al. 2009; Jain et al. 2017; Li et al. 2018). The questionnaire consisted of four distinct sections as follows:

The first section aimed at acquiring sociodemographic characteristics namely; age, gender, educational level, and monthly income. Age was categorised into “20–30”, “31–40”, “41–50”, and “ > 50 years”; while educational level was categorised into primary, preparatory, high school, university, and higher education.

The second section aimed at acquiring information related to parents’ prior knowledge on the management of avulsion, the source of such information, and whether parents were previously directly involved in the management of an avulsion case.

The third section aimed at acquiring information related to parents’ willingness on replanting avulsed teeth at the site of injury and knowledge of different aspects of the emergency management of avulsed permanent teeth. The questions included the ability to differentiate between primary and permanent dentition, the possibility of reinsertion of avulsed teeth, the correct timing to reinsert the tooth, the preferred tooth cleaning options and transportation media, and the best timing and location for seeking professional help. The fourth section aimed at obtaining parents’ opinion as to the best format for future parental education on the management of dental trauma.

Bilingual independent Arabic/English language speakers were involved in forward and backward translations of the original English questions (Hambleton and Zenisky 2010). Furthermore, the questionnaire was piloted on 25 participants to evaluate ease of understanding and appropriateness of the questions. The participants from the pilot study were excluded from the main study sample.

Based on the literature, parental knowledge with respect to emergency management of avulsed permanent teeth in children and adolescents is highly variable ranging from 25 to 60% (Al-Jame et al. 2007; Hashim 2012; Ozer et al. 2012; Alyahya et al. 2018; Alharbi et al. 2020). Therefore, assuming that our cohort had 40% of knowledge with precision of estimate (margin of error) of 5% and 95% level of confidence, a sample size calculation resulted in the need for 370 participants to achieve the objectives of this study. However, the sample size was increased to 400 to account for different subgroup analysis.

The following formula was used in calculating sample size:

n 1⁄4 [Z^2^1–α/2 P(1–P) / d^2^], where n 1⁄4 sample size, Z 1⁄4 *Z* statistic for a level of confidence (for the level of confidence of 95%, which is conventional, *Z* value is 1.96), *P* 1⁄4 expected prevalence or proportion. *d *1⁄4 precision (in proportion of one; if 5%, *d* 1⁄4 0.05).

Descriptive and analytical statistics were conducted. With respect to descriptive statistics, frequency of distribution in relation to demographic data and responses to items of the questionnaire were presented. For analytical statistics, univariate and multivariate logistic regressions were employed to assess the association between the independent variables (sociodemographic status) and knowledge in the management of avulsion. The scoring of knowledge in the management of avulsion was based on the percentage of the correct answers (favourable answers). The percentage of correct answers was calculated by dividing the number of correct answers to the maximum possible number of correct answers multiplied by 100. A percentage of 49 or below was considered poor, 50–69 fair, and > 70 good. To facilitate the regression analyses, the outcomes were dichotomized to either favourable (≥ 50%) or unfavourable answers (˂50%). Independent variables which were significantly associated in the unadjusted regression at (*P* = 0.2) were entered into a final multivariate logistic regression to evaluate their effects after adjustment. The *p*-value was set as 0.05 in the final model, and SPSS 22.0 software (SPSS Inc. Chicago, IL, USA) was used for analysis.

## Results

The dataset comprised data from completed questionnaires of 400 respondents. Most of the respondents were females 60.3% (*n* = 241). Almost half of the respondents 48.3% (*n* = 193) were in the age group category of “31–40” years and with a university educational level 48% (*n* = 192). Of the 400 respondents, 41.8% (*n* = 167) were earning on average “10,000–30,000 Qatari riyals” (2746.50–8239.49 USD) (Table 1).

**Table 1:** The sociodemographic characteristics of the study participants

Gender	*n*	%
Did not answered	3	0.8
Female	241	60.3
Male	156	39.0
Age	*n*	%
Did not answer	13	3.3
> 50	19	4.8
20–30	66	16.5
31–40	193	48.3
41–50	109	27.3
Income	*n*	%
Did not answer	23	5.8
< 10,000	57	14.3
10,000–30,000	167	41.8
30,000–50,000	85	21.3
> 50,000	68	17.0
Education level	*n*	%
Did not answer	8	2
Postgraduate	38	9.5
University basic degree	192	48.0
High school	120	30.0
Preparatory school	25	6.3
Primary school	17	4.3

Almost half of the respondents reported having received information about management of dental trauma 47.3% (*n* = 189). Of those, 28.75% (*n* = 115) stated that they obtained such information from a treating dentist (Table 2). Five respondents (1.25%) reported other sources such as their parents or grandparents. Most of the respondents were not involved in the management of an avulsed tooth (73.5%, *n* = 294) (Table 2).

**Table 2:** The respondents answers about their prior knowledge of dental trauma

	*n*	%
Have you ever received any information regarding traumatic dental injuries?		
No	207	51.8
Yes	189	47.3
Did not answer	4	1
What is your source of information?^*^, ^&^		
Not answered	7	1.75
Booklet	35	8.75
Friend	21	5.25
Educational programme	59	14.75
Dentist	115	28.75
Online resources	58	14.5
Other	5	1.25
Where you ever involved in a situation whereby a child lost a permanent tooth?		
No	294	73.5
Yes	103	25.8
Did not answer	3	0.75

*Out of 189 parents who received an information regarding dental trauma. ^&^Parents were able to choose more than one source

The overall mean score of respondents’ knowledge on the management of dental avulsion was (23%). Most of the respondents 83% (*n* = 332) demonstrated poor knowledge (Correct answers < 50%), while similar number of respondents 8.5% (*n* = 34) demonstrated moderate (Correct answers 50% to < 70%) and good (Correct answers ≥ 70%) level of knowledge 8.5% (*n* = 34).

More than half of respondents 55.8% (*n* = 223) knew the difference in shape between primary and permanent dentition (Table 3). Only 23.3% (*n* = 93) of participants thought that it was possible to reinsert an avulsed permanent tooth. Out of those, only 12.9% (*n* = 12) indicated willingness to reinsert the tooth back into its socket (Table 3). Among the respondents who indicated their willingness to reinsert or maybe they could reinsert, half of them 51.6% (*n* = 22) indicated that it should be done within 30 min (Table 3).

**Table 3:** The respondents answers to the knowledge questions regarding management of avulsed teeth

	*n*	%
Do you know the difference in shape between milk teeth (baby teeth) and adult teeth? ^*^		
No	173	43.3
Yes^#^	223	55.8
Did not answer	4	1
Do you think it is possible to reinsert a tooth that was completely knocked out from a child’s mouth? ^*^		
No	296	74
Yes^#^	93	23.3
Did not answer	11	2.8
Would you personally reinsert knocked out tooth back into its position in the child’s mouth after traumatic injury? ^**^		
No	48	51.6
May be^#^	33	35.5
Yes^#^	12	12.9
If you will reinsert the tooth into its position in the child’s mouth, do you think it should be done? ^***^		
Maximum in 30 min.^#^	22	48.9
Maximum in 1 h.^#^	5	11.1
Maximum in 24 h	2	4.4
I don’t know	16	35.6
If you decide to reinsert the tooth back. What would you do first?***		
Rub the tooth and clean with toothbrush	2	4.4
Rub the tooth with gauze/tissue	6	13.3
Clean under tab water^#^	25	55.6
Not to clean the tooth	11	24.4
Other	1	2.2
If the knocked out tooth is to be reinserted into its position by a qualified professional person, do you think the tooth should be kept in: ^**, &^		
In tissue	15	16.1
In tap water	15	16.1
In Fresh milk^#^	46	49.5
In Saliva^#^	3	3.2
Other	4	4.3
Don’t know	15	16.1
If reinserting the tooth is not possible, when do you think is the best time to look for professional help?^*^		
Maximum 30 min.^#^	111	27.8
Maximum within 1 h.^#^	74	18.5
Maximum within few hours	68	17
After 1 day	56	14
Any time	56	14
Did not answer	35	8.8
Where would you take a child after dental injury, which caused completely knocking out tooth? ^**,&^		
Call emergency department	5	5.3
A dentist in Primary Health Center^#^	41	44
Emergency department in governmental hospital^#^	52	56
Private dental centre^#^	18	19.3
Will not go anywhere	1	1.1

^*^Out of all parents (*n* = 400), **Out of 93 parents who thought an avulsed tooth can be replanted, ***Out of 45 parents who would personally reinsert a tooth back into its socket. ^&^parents were able to choose more than one media and more than one option in regard to where to take the child after dental injury. ^#^Correct answer

Out of those who believed an avulsed tooth could be replanted 23% (*n* = 93), regardless of whether they would reinsert the tooth or not, almost half 48.4% (*n* = 49) indicated that an avulsed tooth should be stored using physiological media. Of note, 16.1% of respondents did not know which media the teeth be stored into (Table 3).

Furthermore, almost more than half of the respondents (56%) indicated that they would then take the child to a hospital emergency department, while around 44% would visit a dentist at a primary health centre (Table 3). With regards to the best timing to look for professional help following avulsion, less than one third (27.8%) indicated that they would seek professional help within 30 min (Table 3).

**Fig. 1:**
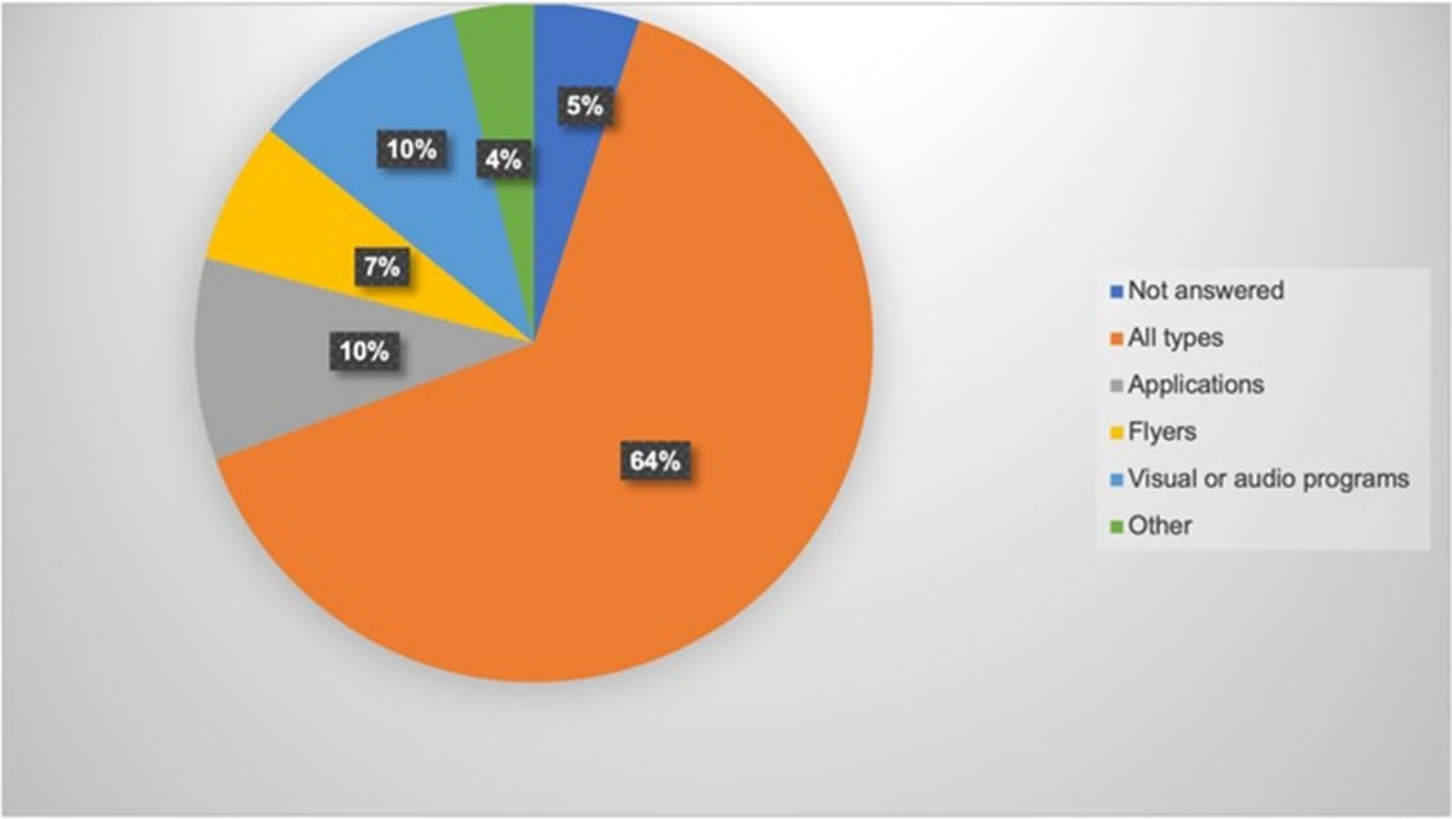
Pie chart showing parent’s preferred way of communication with regards to spreading awareness with regards to emergency management of dental avulsion

With respect to the best way to spread awareness about how to deal with dental injuries, most respondents (64%) chose all the suggested forms of communication (Fig. 1). Of the remaining participants who chose one form of communication, most were in favour of visual/audio programmes 10% or mobile phone applications 10% (Fig. 1).

According to the unadjusted univariate logistic regression, gender and age were the only sociodemographic variables that were statistically significant at the 0.2 level (Table 4). Males were less likely to give favourable answers when compared to females (OR = 0.6, 95% CI = 0.3–1.04). Age groups “31–40” years and “41–50” years were more likely to give favourable answers when compared to 20–30 years age group (OR = 2.1, 95% CI = 0.9–5.4 and OR = 2.8, 95% CI = 1.1–7.3; respectively; Table 4).

**Table 4:** The results of the univariate logistic regression and adjusted multivariate logistic regression used to assess the association between the independent variables (sociodemographic status) and knowledge in the management of avulsion showing odds ratios (OR), and 95 per cent confidence intervals (95% CIs)

Predictor variables	^a^Univariate logistic regression		*P*-value	^b^Multivariate logistic regression	
	Percentage (%) of positive knowledge score	Unadjusted odds ratio (OR) and 95% CI		Adjusted odds ratio (OR) and 95% CI	*P*-value
Gender					
Female	60.3	1.0 (Reference)		1.0 (Reference)	
Male	39	0.6 (0.3–1.04)	0.069	0.43 (0.22–0.84)	0.013
Age group					
20–30 years	16.5	1.0 (Reference)		1.0 (Reference)	
31–40 years	48.3	2.1 (0.9–5.4)	0.1	2.8 (1.05–8.0)	0.05
41–50 years	27.3	2.8 (1.1–7.3)	0.03	3.8 (1.3–11.48)	0.015
> 50 years	4.8	1.9 (0.4–8.3)	0.41	7.7 (0.89–42.61)	0.18
Income					
< 10 K	14.3	1.0 (Reference)		1.0 (Reference)	
10–30 K	41.8	0.56 (0.26–1.24)	0.25	0.36 (0.14–0.86)	0.23
30–50 K	21.3	0.87 (0.37–2.01)	0.74	0.52 (0.20–1.35)	0.18
> 50 K	17	0.97 (0.4–2.3)	0.95	0.54 (0.2–1.5)	0.23
Education level					
Preparatory	6.3	1.0 (Reference)		1.0 (Reference)	
Primary	4.3	0	0.99	0	0.99
High school	30.0	1.46 (0.4–5.4)	0.56	1.53 (0.37–6.32)	0.55
University	48.0	1.5 (0.4–5.3)	0.5	1.33 (0.3–5.43)	0.68
Post graduate	9.5	2.6 (0.6–10.68)	0.18	2.11 (0.43–10.42)	0.35

^a^Simple logistic regression was conducted between each sociodemographic variable and the outcome variable. *P*-value of ≤ 0.2 was considered significant. ^b^Multiple logistic regression was conducted. Explanatory variables that were significant at the 0.2 level from the simple logistic regression were entered in the final model after assessing multicollinearity. *P*-value < 0.05 was considered significant in the final model

As a result, gender and age groups were entered into the final multivariate logistic regression model after adjusting for the other sociodemographic variables that were not statistically significant in the unadjusted univariate analysis (Table 4). According to the final model, gender and age groups “31–40” years and “41–50” years remained statistically significant predictors and this was at the 0.05 level (*P* = 0.01, *P* = 0.05 and *P* = 0.01; respectively).

## Discussion

Dental avulsion is one of the few time-sensitive urgent emergencies in dentistry whereby urgent onsite management can make the difference between tooth retention or a lifelong dental burden. The prognosis for avulsed teeth is improved by prompt and appropriate treatment, which often depends upon the knowledge of those present at the site of an accident before professional dental care can be provided (Andreasen et al. 2019). Lack of knowledge on the appropriate onsite urgent management could result in no implantation of avulsed teeth or the use of inappropriate storage media prior to replantation. Such steps can severely reduce the prognosis of the treatment provided leading to tooth loss. Parents are among the first responders of such tragic injuries; therefore, assessment of their knowledge and factors affecting their knowledge on the management of such severe injury is crucial. To the author’s knowledge, this is the first study assessing the level of parental knowledge and awareness with respect to the management of avulsed teeth in the State of Qatar. The high human development index and high per capita income of the State of Qatar renders this country unique among neighbouring Gulf Corporation Council (GCC) and Middle Eastern Countries, therefore warranting assessment of parental knowledge and factors affecting such unique country.

The results of this study showed that the overall knowledge of Qatari parents was towards the lower end of those reported by neighbouring countries with similar sociodemographic characteristics to that of the State of Qatar, such as United Arab Emirates (16.6%), Kuwait (37.5%), Saudi Arabis (47.7%) (Hashim 2012; Alharbi et al. 2020), and international studies in UK (19%) (Walker and Brenchley 2000) and Australia (24.7–45%) (Raphael and Gregory 1990). Although almost half of the respondent’s reported obtaining previous information on the management of avulsion, such information did not improve their level of knowledge as shown in this study. Such trend is evident in other studies where previous knowledge on the management of general dental trauma did not correspond with the respondents’ actual current knowledge (Quaranta et al. 2014; Świątkowska et al. 2018). These results might highlight the importance of having readily accessible trauma management information in the form of mobile applications where parents/lay people can obtain onsite guidance rather than rely solely on their recollection of information.

Parental misinformation on the importance and possibility of replanting avulsed teeth, which is in line with other published studies (Alyahya et al. 2018; Alharbi et al. 2020), is of great importance and consequence. Such misinformation could lead to parents leaving avulsed teeth at the site of trauma or dispose of these teeth which would unnecessarily compromise their children’s dental health. Furthermore, parents’ lack of differentiation between primary and permanent teeth and lack of willingness to replant the tooth at the site of trauma would compromise the success of such cases. Unfortunately, parental lack of awareness the need for urgent replantation either onsite or by dental professionals within 30 min, and best suitable storage media can compromise the success and survival of such avulsed teeth which also highlights the importance of parental education and/or access to onsite first aid information. The extra alveolar dry time of an avulsed tooth and the storage media are critical factors that affect periodontal ligament cells (PDL) viability and the outcome of the implantation (Fouad et al. 2020). At an extra alveolar dry time of 30 min, most of PDL cells would become non-viable (Andreasen 1981; Barbizam and Massarwa 2015). This study highlighted parental lack of knowledge on the most suitable storage medium which is also in line with published studies in neighbouring (15% in Kuwait, 27% Saudi Arabia) and international (5% in Australia and 29% in UK) countries. The urgency of replanting avulsed teeth was assessed in the present study through two questions. The first tackled parents’ knowledge on whether time was a crucial factor in replating the avulsed tooth while the other assessed their knowledge of the urgency of contacting a dental professional for urgent replantation of the avulsed teeth. Only one study (Alharbi et al. 2020) assessed parents’ knowledge of the urgency of replanting avulsed teeth rather than the speed of contacting dental professionals which found that 26.5% of parents were aware of the importance of replanting the tooth within 30 min of the avulsion compared to only 5.5% (out of all participants) in the present study.

Both parents’ age and gender were found to be statistically significant predictors of parent’s knowledge on the management of tooth avulsion, whereby older parents and mothers showed better knowledge. This is probably because mothers, in the State of Qatar, are generally more involved in their children’s upbringing.

Although parents were well educated in this study, with the majority having a university educational level, parental education had no influence on the knowledge of tooth avulsion management. In contrast, parents’ education was significantly associated with parental knowledge in Saudi Arabia, while it was not in Kuwait (Alyahya et al. 2018; Alharbi et al. 2020). Similarly, parents’ income showed no correlation with parents’ knowledge in line with the study performed in Kuwait. The high income per capita in the gulf region, especially the State of Qatar, might have masked such influence. The majority of respondents in this study reported an income above 10,000 Qatari Riyals (around 2700 USD) per month.

In terms of seeking professional help, the current study has highlighted severe lack of parental knowledge in terms of the need to urgently seek professional dental help which is much lower than that reported in Kuwait (74.9%) and UK (80%) (Walker and Brenchley 2000; Alyahya et al. 2018). Of concern, a large proportion of the parents in the present study chose to take their children to a hospital emergency department rather than a dental clinic where urgent replantation of teeth by the medical team might not be possible. The emergency services in the State of Qatar, as in most countries around the world, lack trained medical staff able to deal with such dental emergency and where waiting times range between 3.8 and 19.2 h (Siamisang et al. 2020; Al Nhdi et al. 2021). Data from a UK-based study, whereby the knowledge of emergency medical staff on the management of avulsed teeth was assessed, showed critical lack of knowledge despite a third of the respondents having prior training on the management of avulsed teeth. Surprisingly, only 15% of those medical staff reported that they would advise parents to replant the tooth, 33% to contact a dentist, and 21% to place in milk (Walker and Brenchley 2000).

Interestingly, most of the participants in this study expressed their desire to increase their knowledge and benefit from all available means of education on the management of dental avulsion such as, flyers, visual, or audio programmes, and application for smartphones. Further investment in spreading awareness on the management of dental emergencies, including dental avulsion, is crucial among lay people and frontline medical professionals. In recognition of the importance of having reliable onsite information on the management of dental emergencies, the International Association of Dental Traumatology has developed a free mobile phone application targeting parents and professionals alike (https://www.iadt-dentaltrauma.org/for-patients.html). This application is available in 9 languages (English, German, Greek, Hebrew, Italian, Polish, Spanish, Turkish, and Chinese). Translation of this application and other IADT resources into Arabic language would help lay people in the Arabian region better manage dental emergencies. Furthermore, dental authorities should support such initiatives and help inform parents, teachers, and medical professionals of the availability of such onsite tools.

In addition, investing in emergency medical staff training on the management of dental emergencies is crucial to improve emergency care for patients suffering from dental trauma. Moreover, providing access to maxillofacial or dental professionals at emergency departments would accelerate receiving the appropriate intervention and consequently improve the prognosis.

This study was not without limitations, especially related to the source of recruited parents. Recruitment of parents attending dental appointments at specialty governmental service might have affected the results of this study as such parents might have witnessed dental trauma and been informed of the appropriate management of avulsion which could have resulted in an underestimation of parental knowledge.

## Conclusion

Within the limitations of this study, the results highlight critical deficiencies in parental knowledge on the management of tooth avulsion which is likely to compromise the long-term success of these teeth leaving children with a lifelong dental burden. Not only does this study highlight the need to improve parents’ knowledge on the management of dental emergencies, but also of the need for developing easily accessible onsite emergency management tools.
